# Settling for Less: Do Statoliths Modulate Gravity Perception?

**DOI:** 10.3390/plants9010121

**Published:** 2020-01-18

**Authors:** Franck Anicet Ditengou, William David Teale, Klaus Palme

**Affiliations:** 1 Institute of Biology II, Faculty of Biology, Albert-Ludwigs-University of Freiburg, Schänzlestrasse 1, 79104 Freiburg, Germany; 2 BIOSS Center for Biological Signaling Studies, Albert-Ludwigs-University of Freiburg, Schänzlestrasse 18, 79104 Freiburg, Germany; 3 State Key Laboratory of Crop Biology, College of Life Sciences, Shandong Agricultural University, Daizong Street 61, Tai’an 271018, China; 4 Sino-German Joint Research Center on Agricultural Biology, College of Life Sciences, Shandong Agricultural University, Daizong Street 61, Tai’an 271018, China

**Keywords:** gravity, root gravitropism, *Arabidopsis thaliana*, statolith, microgravity

## Abstract

Plants orientate their growth either towards (in roots) or away from (in shoots) the Earth’s gravitational field. While we are now starting to understand the molecular architecture of these gravity response pathways, the gravity receptor remains elusive. This perspective looks at the biology of statoliths and suggests it is conceivable that their immediate environment may be tuned to modulate the strength of the gravity response. It then suggests how mutant screens could use this hypothesis to identify the gravity receptor.

## 1. Introduction

Responsive growth is a feature of land plants. Diverse environmental inputs are prioritized and integrated into a single output: cell elongation in specific regions of the plant. This differential growth is characteristic of the gravity response and involves the spatial separation of sensing and expanding cells. Such separation requires the presence of a mobile and responsive signaling pathway, which is able to convert physical strain into a physiological response. The directional transport of auxin is a prominent mobile component of this pathway, but although many of its transporters and molecular targets have been identified, important features of the wider signaling environment remain unknown [1].

## 2. Auxin as a Mobile Signal

Direct evidence for the involvement of auxin transport in the gravitropic response has come from two complementary strands of research: firstly, from the observation that an alteration in a plant’s capacity for auxin transport can, depending on the context of the change, lead to a decrease in its ability to respond to changes in the gravity vector. This is most clearly seen after the application of auxin transport inhibitors but is equally obvious in genotypes which lack key auxin transport proteins such as AUX1 and PIN2 [2,3], or in specific auxin signaling mutants, such as *axr2*/*iaa7*, which are unable to translate an auxin signal into a growth response [4]. In addition, the effect of auxin activity on root gravitropism is modulated by cross talk with other plant hormones such as ethylene, cytokinins or gibberellins [5,6,7,8,9,10]. Recently, we showed that the spatial distribution of the folate precursor *para*-aminobenzoic acid (*p*ABA) is under the control of a UGT74B glucosyltransferase, which regulates auxin-ethylene cross talk and is necessary for the gravitropic response in roots [11]. The second line of evidence for the involvement of auxin transport in the gravity response follows the observation that auxin redistribution occurs immediately before bending begins [3,12].

## 3. The Statolith

At the root tip, columella cells occupy the central part of the root cap and are characterized by the polar organization of their organelles [13]. The maintenance of this structural asymmetry of the cell relies on microtubules and the actin cytoskeleton [13,14]. In columella cells, the nucleus and the endoplasmic reticulum are located at the opposite ends of the cell and microtubules at the periphery. Despite recently being called into question [15,16], it is widely held that in roots, gravity is perceived in these columella cells by the sedimentation of starch-filled amyloplasts called statoliths. Statoliths, denser than the cytoplasm, occupy the center of the cell and are packed in a dense mesh of actin filaments (AFs) which links them both to each other and to the plasma membrane [13,17]. The gravity-driven redistribution of statoliths stimulates a signaling cascade which is initially cell-autonomous but results in the cell-to-cell asymmetric movement of auxin and a directional growth response [18]. However, as previously noted, both the identity of the mechano-receptors that are hypothesized to sense statolith sedimentation and its immediate signaling cascade are unknown [19].

Although the word statolith literally means motionless stone, statoliths are highly mobile. In maize root cap cells and away from the root in the endodermal cells of a coleoptile, a group of statoliths have been shown to exhibit saltatory (or jumpy), F-actin-dependent non-Brownian movements [20,21,22]. The amplitude and the speed of these movements vary with the type of organ studied, suggesting that intracellular environments surrounding statoliths might affect the sensitivity of the root to gravity. The expression of *PHOSPHOGLUCOMUTASE 1* (*PGM1*) as well as two other starch granule biosynthesis genes *STARCH SYNTHASE 4* (*SS4*) and *ADP GLUCOSE PYROPHOSPHORYLASE 1* (*ADG1*) is under the control of auxin, with auxin signaling mutants also showing defects in statolith structure [23]. It has been noted that auxin is therefore likely to control not only the amplitude and direction of the growth response, but also a plant’s sensitivity to an initial gravity stimulus [24].

Here, we must emphasize that not only is the identity of the gravity perception apparatus unknown, but even whether or not gravity is sensed by a single protein receptor is also an open question. However, we surmise that it is, and the responsiveness of plants to gravity is, at least in part, regulated by its expression. This expression may be regulated in response to a high heterogeneity in the relative gravity vector (as may be experienced when a stem grows in gusty winds, or a root grows through a stony substrate). In this case, it is conceivable that an auxin-mediated negative feedback mechanism between statolith sedimentation rates and receptor abundance or sensitivity may be a component of the response to gravity. If this were the case, one could expect that genes encoding gravity receptors or their regulators might be among the earliest genes whose expression is regulated after gravity perception. It is therefore possible that suppressors of mutant phenotypes conferring an increased sensitivity to statolith sedimentation effect the regulation of signal transduction very close to a mechanosensitive receptor or the receptor itself.

## 4. Statoliths and the Vacuolar Membrane

In addition to auxin-dependent gene expression, the structure of the vacuole also has a profound effect on statoliths sedimentation dynamics; in the shoot, disrupting a SNARE protein functioning in vacuole biogenesis severely restricts statoliths movement in endodermis cells before and after gravistimulation by reorientation [25]. The vacuolar membrane is important in this context as it is likely to enclose statoliths along with a thin layer of cytoplasm [26]. It therefore must be borne in mind that it is theoretically possible for a putative receptor to also be localized to the vacuolar membrane and not solely to the plasma membrane.

A recent study by Bérut et al. [27] showed that statolith sedimentation in gravistimulated endodermal cells quickly responds even to small changes in bending angle. Statoliths therefore behave less like a pile of stones and more like a liquid. It has been hypothesized that cell-generated active (non-Brownian) fluctuations strongly modulate statoliths movements to achieve this effect. Cytoskeletal network dynamics are good candidates for modulating this agitation [27]. Though its regulation has not been linked to changes in the sensitivity of gravitropic responses, it seems possible that local changes in cytoplasmic streaming affect statoliths sedimentation rates.

The characterized mutants which have been placed furthest upstream in the gravity response, are thought to prevent the stimulation of a receptor by hindering statolith sedimentation, either by physical impediment, or by reducing statolith weight. Statolith sedimentation rate depends on factors such as their number, size and density, as well the strength with which they interact with actin filaments. Accordingly, starch-deficient mutants such as the *pgm* series display a significant reduction in root and shoot gravitropic responses. In line with this observation, the amplitude of the gravity response has been shown to be proportional to the amyloplasts’ starch content across several genotypes [28,29,30,31]. *SHOOT GRAVITROPISM2* (*SGR2*) encodes a phospholipase A1-like protein which localizes to the statolith-surrounding vacuolar membrane in the shoot and in the root [26]. Its activity in the shoot endodermis modulates the hypocotyl and stem gravity response by physically preventing statolith sedimentation. Tellingly, statolith sedimentation and gravitropic curvature can be rescued in *sgr2* (and other floating-statolith mutants) by exposing plants to hypergravity. This is not the case for agravitropic downstream signaling mutants such as the auxin signaling mutant *short hypocotyl 2* (*shy2*) [32]. Similarly, *sgr4* plants display an impaired gravitropic response, which can also be attributed to induced changes in vacuole morphology. Interestingly, the gravity response in *pgm1*, *sgr2* and *sgr9* mutants (which all display abnormal statoliths distribution at 1*g*) could be restored when plants were grown in a centrifuge under hyper *g* conditions, suggesting that hypergravity triggered a functional gravitropic mechanism that is inactive at 1*g* [32]. As artificially increasing statoliths weight in this way rescued specific agravitropic phenotypes, it is likely that the gravireceptor and downstream signaling mechanism remained unimpaired. The agravitropic phenotype of *shy2*, in contrast, is caused by a genetic lesion in pathways downstream of the initial signal perception. *Sgr2, 3* and *4* roots show no agravitropic phenotype, possibly as vacuolar membrane distribution has been seen to differ in shoot endodermal and root collumella cells [22]. The lack of a gravitropic response in stems of *sgr4*, even under hyper *g* may be associated with the fact that the vacuolar membrane does not surround statoliths in this mutant [22].

## 5. Statolith Movement

In the gravistimulated Arabidopsis inflorescence stem of *sgr9*, statoliths in endodermal cells do not sediment but form clusters that are strongly linked to actin filaments. This attachment increases statolith saltatory movement [33]. The gravitropic response in *sgr9* is restored when the integrity of actin filaments is impaired either genetically (such as when crossed into the *fiz1* genetic background (containing an *act8* semidominant mutation that induces the fragmentation of actin microfilaments) or chemically (using Latrunculin B, an inhibitor of actin filaments polymerization). These data suggest that statoliths are in equilibrium between sedimentation and saltatory movement in wild-type endodermal cells [33]. In line with this hypothesis, SGR9 was identified as a RING-type E3 ligase which localizes to the surface of statoliths, where it mediates the statolith-AF interaction [33], promoting detachment and allowing statoliths to sediment with gravity [33]. All together these observations suggest the existence of a graviperception machinery which involves the interaction between statolith-anchored proteins and actin filament-interacting (or associated) proteins. The gravity-triggered translocation of PIN3 from one cell side to another is also actin-dependent in columella cells, lending credence to this hypothesis [34,35]. It is interesting to note that mammalian orthologs of SGR9 are involved in membrane trafficking [36,37,38]; whether SGR9 affects transcytosis of PIN3 remains to be assessed [35]. The missing link between statolith sedimentation and auxin distribution seems to involve the *LAZY1* family genes (reviewed by Nakamura and colleagues [39]). Interestingly, gravity response is perturbed in *lzy* mutants whilst statolith sedimentation occurs normally, suggesting that the *LZY* genes mediate gravity signaling downstream of statolith sedimentation [40]. Phototropic responses are unaffected indicating that asymmetrical auxin redistribution and auxin-responsive growth are unaffected and that the defect lies upstream of these processes in a gravitropism-specific branch of the pathway. LAZY is therefore hypothesized to be a bridge which connects gravity perception and auxin re-distribution. LAZY itself is a protein of unknown function; with a plasma-membrane-associated localization but containing a transcriptional-repressor-binding EAR motif, its biology is set to puzzle physiologists for a long time to come [41].

## 6. Outlook

The strength of the gravity signal is modulated at multiple points, some of which affect the weight of statoliths, whilst others affect the sensitivity of the responsive signaling components. Under what circumstances this modulation occurs in nature is as yet unclear, as is the selective advantage modulation of gravitropic signaling may confer. However, as biologists, we know that nature rarely passes up an opportunity to regulate and control. A wide range of techniques and resources are currently available to researchers, including clinostats, sounding rockets, parabolic flights and even prolonged exposure to micro-*g* on the International Space Station. Here, valuable insights are already contributing to our understanding; for example, in parsing the hierarchy of tropic responses to different stimuli by comparing growth responses on the ISS with those on the ground (gravitropism trumps chemotropism and hydrotropism) [42]. It will be important now to explore the boundaries of these relationships; for example, as salt concentrations increase, which signaling pathways determine that a halotropic response should take precedence over a gravitropic one [43]? A point of interaction between gravitropism and abiotic stresses is the regulation of statolith structure by for example gradients of either moisture [44] or NaCl [45]. The ISS has also been involved in elucidating the role of transient increases in cytosolic calcium ion concentration, where very small changes in statolith positioning are sufficient to trigger measurable changes in concentration [46]. These experiments built on an influential report which used pharmacological treatments to manipulate cytosolic calcium ion concentration to demonstrate the close functional links among cytoplasmic calcium ions, statolith integrity and the gravitropic response [47].

Questions as to the extent to which amyloplast content influences the gravitropic response in diverse land plant phyla (for example in the pteridophytes) have been recently raised, opening promising comparative experimental systems [24]. This is particularly interesting as alternative gravity perception mechanisms which do not involve statolith sedimentation have been proposed to explain gravity-responsive growth in organisms such as fungi, algae and mosses (see [48,49,50]). For example, the gravitational compression model posits that gravity is sensed though the settling of the whole mass of the protoplast on the extracellular matrix [51,52]. In this case, all cell organelles (including statoliths) would function as makeweights to increase the sensitivity of the cell to gravity [51]. On the other hand, the tensegrity model depends on the interaction between the organelles and the cytoskeletal array (AFs and microtubules). Upon gravistimulation, due to their weight, organelles might deform the cytoskeleton mesh which surrounds them to generate a modest force but one large enough to influence the properties of cellular proteins [53,54]. Whether these alternative mechanisms intersect with those modulating the perception of statolith displacements remains an open question. However, it is noteworthy that all of these mechanisms are not mutually exclusive and may operate simultaneously in gravistimulated cells.

**Figure 1:**
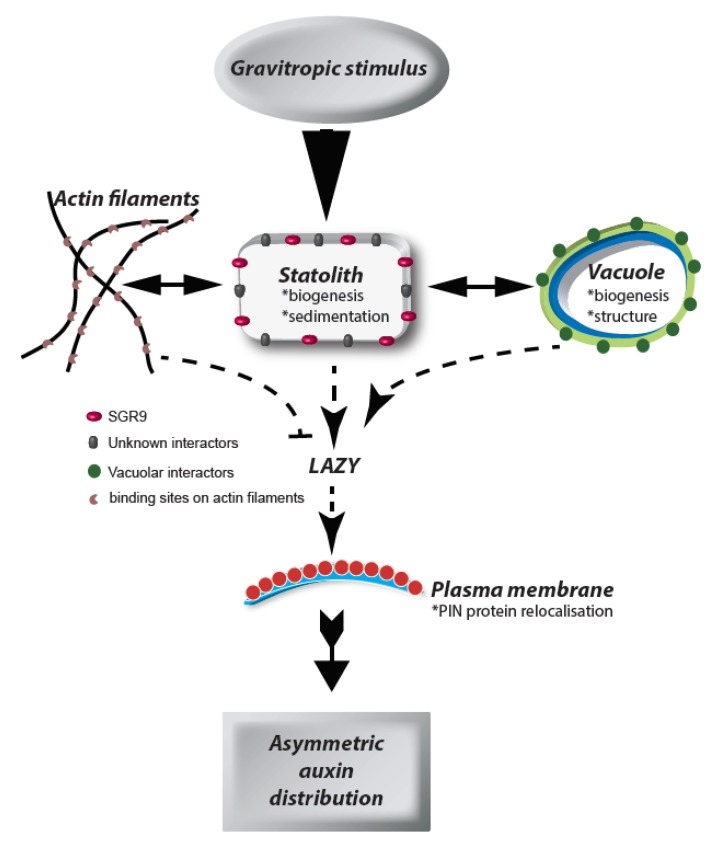
Graviperception flowchart. Graviperception is mediated by statolith displacement in root columella cells. Statoliths interact with the vacuole and actin cytoskeleton, respectively, via proteins on their surface. Interaction with actin delays the graviresponse whereas interaction with membranes triggers re-localization of PIN-proteins via LAZY and subsequent asymmetric auxin distribution, which inhibits growth on the lower side of the root.

Here, we have expounded the perspective that the elusive identities of upstream signaling components, or even the gravity receptor itself, may be uncovered by the search for pathways which regulate the sensitivity of the gravitropic response (Figure 1). For example, the expression of which genes is downregulated under a pABA-induced hypergravitropic response? Is the expression of these same genes upregulated in light-statolith agravitropic mutants? Does gene expression analysis after exposure to different gravity regimes also show differential regulation of these same transcripts? Mutant suppressor screens are also likely to be informative when aimed at identifying genotypes which suppress insensitivity to gravity. It is, however, becoming increasingly clear that an ambitious combination of next-generation sequencing and dynamic network analysis after growth responses induced under different gravity regimes may have the capacity to identify the gaps in our understanding of gravity perception.
